# Safety of Live Attenuated ASFV-G-ΔI177L/ΔLVR Vaccination in Sows With Advanced Pregnancies

**DOI:** 10.1155/tbed/8007143

**Published:** 2025-07-13

**Authors:** Su Jin Lee, Yeonji Kim, Sun A. Choi, Keun Seung Ahn, Se Young Lee, Xinghua Zheng, Do Soon Kim, Wonjun Kim, Yongwoo Shin, So-Jeong Kim, Sua Choi, Dongseob Tark, Seong Cheol Moon, Weonhwa Jheong, Jung Hyang Sur

**Affiliations:** ^1^Central Research and Development Institute, Komipharm International Co., Ltd., 17 Gyeongje-ro, Siheung-si, Gyeonggi-do 15094, Republic of Korea; ^2^National Institute of Wildlife Disease Control and Prevention Wildlife Disease Response Team, Ministry of Environment, Songam-gil 1, Gwangsan-gu, Gwangju 62407, Republic of Korea; ^3^Laboratory for Infectious Disease Prevention, Korea Zoonosis Research Institute, Jeonbuk National University, 820-120, Hana-ro, Iksan 54531, Republic of Korea

**Keywords:** African swine fever, live attenuated vaccine, piglets, pregnant sows, protective efficacy, safety, vertical transmission

## Abstract

African swine fever (ASF) causes severe disease in domestic pigs and wild boars and often results in 100% mortality. The use of attenuated strains in ASF vaccine development, based on the most recently studied ASF virus (ASFV)-Georgia (ASFV-G) strain, has proven effective. In July 2023, Vietnam became the first country in the world to have two indigenously developed ASF vaccines approved for commercial sale locally. In this study, we evaluated the immunogenicity and safety of the live attenuated vaccine (LAV) ASFV-G-ΔI177L/ΔLVR derived from ASFV-G-ΔI177L in five pregnant sows and a control group. The ASFV-G-ΔI177L replicates efficiently in a stable swine cell line. Therefore, the pregnant sows were used to validate the vaccine. Fundamental assessments of clinical symptoms (vaccine safety), parturition performance, vertical transmission presence after birth, and maternal antibodies were conducted before and after colostrum intake following the vaccination with ASF-G-ΔI177L/ΔLVR. The vaccination had no adverse effects and induced a high level of antibody production. Normal delivery was also confirmed without miscarriages. Furthermore, ASF antibodies and the P72 gene were detected in the blood of most piglets that consumed colostrum from vaccinated sows, and transplacental transmission was confirmed in piglets following vaccination. While these results will influence vaccine development and play an important role in preventing lethal ASFV infections, they also highlight the potential of ASFV-G-ΔI177L/ΔLVR to protect even high-risk groups such as pregnant sows and emphasize the importance of continued vigilance and investigation of vaccine safety parameters such as viral shedding, genetic stability, and reproductive efficacy.

## 1. Introduction

African Swine Fever (ASF) is a highly lethal swine disease that can affect both farm-raised and wild pigs. It manifests in various forms, ranging from very severe to subclinical infections, which are affected by various factors [1]. ASF is caused by the ASF virus (ASFV), which belongs to the family Asfarviridae and genus *Asfivirus*. ASFV is a large icosahedral virus that contains a linear double-stranded DNA genome (170–190 kbp) encoding approximately 165 proteins. Twenty-five genotypes have been identified with varying virulence [2–5]. In continents endemic to ASF, its incidence among domestic pigs and wild boars is gradually increasing. As of January 2024, some Balkan countries in Europe had reported a significant increase in ASF outbreaks in domestic pigs throughout the summer and fall. Some countries, such as Italy, Latvia, and Poland, reported an increase in cases affecting wild boars through the winter months in less than a year [6]. In 2018, ASF was first reported in China and has since spread to Southeast and Northeast Asia, with countries such as Vietnam, the Philippines, Laos, and South Korea experiencing more severe outbreaks with major consequences for the pig industry [7].

There is no commercial vaccine or antiviral agent to control the more than 25 different genotypes of ASFV. However, attempts to produce live attenuated vaccines (LAVs) are underway. These are primarily focused on deleting specific genes from ASFV to reduce virulence in pigs without interfering with efficient growth in cell culture [8, 9]. One such vaccine, ASFV-G-ΔI177L, has demonstrated satisfactory efficacy against the Genotype II ASFV strain from Georgia 2007, the parental virulent strain, and other regional outbreaks [10]. After a thorough safety evaluation, this vaccine was approved for commercialization in Vietnam, marking a global first [11].

Previously, the ASFV-G-ΔI177L/ΔLVR vaccine candidate was thoroughly tested in domestic pigs for immunogenicity, safety, and protection against highly virulent field strains [9, 12]. For further analysis in this study, experiments were conducted on pregnant sows under defined conditions. The key objectives of these experiments were to confirm the safety and presence of vaccine antibodies in sows at the end of gestation, as well as the formation of maternal antibodies from colostrum and the transmission of the ASF vaccine strain through the placenta.

## 2. Materials and Methods

### 2.1. Cell Culture and Virus

The production of the master seed virus (MSV) based on cell culture requires ease of cultivation, genetic stability during passaging, and high virus yield. Adherence to quality standards is crucial, as outlined by the World Organization for Animal Health (WOAH) Biological Standards Committee Annex 32 (Chapter 3.9.1., African swine fever: Infection with African swine fever]) [13]. To characterize the biological properties of MSV, the following procedures were implemented. The vaccine candidate ASFV-G-∆I177L/∆LVR is a cell culture-adapted vaccine virus, which is the eighth (Passage 8, P8) generation of the PIPECs (Plum Island porcine epithelial cells) line [9] from the USDA. Since the release, another passage was added to produce the ninth passage (P9). Subsequently, 10 further passages yielded P19. This exhibited the highest vaccine titer selected as MSV (ASFV-G-ΔI177L/ΔLVR-P19) after confirming various genetic modifications. Pregnant sows were immunized with the ASFV-G-ΔI177L/ΔLVR (P19) vaccine candidate.

### 2.2. Animal Experiments

Animal experiments were conducted under animal biosafety level 3 (ABSL-3) conditions at the Animal and Plant Quarantine Agency using a protocol approved by the Institutional Animal Care and Use Committee (IACUC; Protocol APQA 2024-820). According to the experimental design, we planned to use a minimum of 10 pregnant pigs for the initial vaccination trial. However, we were forced to use five pregnant pigs due to the limited space in the ABSL-3 facility of the Korean government and the limited time allowed for the trial. These limitations of the ABSL-3 was also the reason why the pigs were vaccinated after 3 instead of 7 days of acclimatization to the new environment.

Five sows that had been pregnant for 90–95 days (final stage of pregnancy) were used in this study. The five pregnant sows were selected from healthy late gestation sows of similar age (average 5–6^th^ postpartum) from the same farm. Previous farrowing history according to calving date is detailed in Table S4. Of these, three were assigned to the vaccination (V) group, and the remaining two were assigned to the control (C) group. The ASF vaccine used in the pregnant sow experiments was the ASFV-G-ΔI177L/ΔLVR candidate, which was tested against ASFV, porcine reproductive and respiratory syndrome virus (PRRSV), foot-and-mouth disease virus (FMDV), porcine circovirus 2 (PCV2), classical swine fever virus (CSFV) and mycoplasma with antigen negativity and antibodies to each disease confirmed before vaccination. PCR and enzyme-linked immunosorbent assay (ELISA) tests for prescreening of major swine diseases can be found in Tables S1–S3.

All experimental animals were divided into two groups for intramuscular immunization (IM). The pregnant sows received IM with 1 mL of ASFV-G-ΔI177L/ΔLVR with 10^3.5^ TCID_50_ (Group V). The control group were administered a mock vaccine (Group C; Table 1).

**Figure 1:**
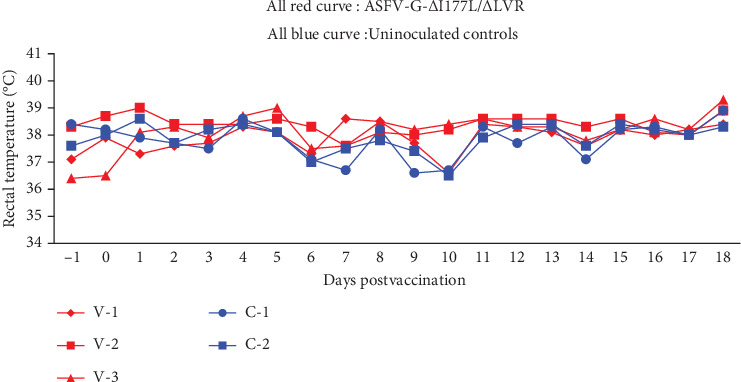
Rectal temperature of the pregnant sows administered IM with 10^3.5^ TCID_50_ of ASFV-G-ΔI177L/ΔLVR. Rectal temperature (*y*-axis) for each group of pregnant sows following inoculation. The date of inoculation is shown on the *x*-axis.

The sows were divided into two groups after they were housed, allowed to stabilize over 3 days and vaccinated on the fourth day. Unlike normal growing pigs, the pregnant sows were not subjected to frequent blood sampling and nasal and rectal swabbing. Therefore, samples were collected for the ASF vaccine DNA (ASFV P72 gene) and antibody confirmation on Day 11 postvaccination (11 dpv) and Day 20 when all farrowing was completed (20 dpv). The clinical signs and rectal temperature were recorded daily during the experiment (Figure 1). To synchronize the births of the pregnant sows, labor was induced (LUTALYSE; dinoprost tromethamine, 5 mg/mL, Zoetis, Korea) on the eighteenth day after vaccination, and all sows were evaluated for farrowing performance on Days 19–20 (Table 2).

Each piglet underwent health assessments and was further tested for ASFV P72 gene and antibodies after farrowing. Five healthy piglets that had consumed colostrum for at least 48 h from vaccinated sows and five piglets that had not (a total of 10 piglets per vaccinated sow) were tested for ASFV P72 genes (whole blood) and antibodies (serum) after their blood draw.

In addition, a group of piglets (V1-1–4) without colostrum intake from a specific sow (V1) was selected and euthanized to confirm pathological findings. Major organs were collected and placental transmission was confirmed via a quantitative polymerase chain reaction (qPCR) assay (Table 3). The remaining animals were euthanized after the completion of all experiments in strict compliance with research ethics regulations.

### 2.3. Quantitative Real-Time PCR (qPCR) Protocol for the Detection of the ASFV Genome

qPCR was used to detect the virus in the whole blood, swabs, colostrum, and tissue samples at vaccination. The ASFV DNA was isolated using the DNeasy blood kit (Maxwell RSC Whole Blood & RSC Tissue DNA Kit, Promega, USA) and equipment (Maxwell CSC 48 Instrument IVD, Promega, USA) according to the manufacturer's instructions. The methods of DNA purification from whole blood (WB) and swab and tissue samples are similar. For WB and swab samples, 30 µl of Proteinase K solution is added to each incubation tube and 300 µl of WB and swab samples, respectively. Additionally, 300 µl of lysis buffer is added to each incubation tube. Each tube is vortexed for 10 s and incubated in the heating block (set to 56°C) for 20 min. The DNA purification method for tissue samples is as follows. Up to 25–50 mg of tissue sample is weighed and placed in a microcentrifuge tube and homogenized using ceramic bead tube. After that, 80 µl of TE buffer is added to the sample in the tube and each tube is vortexed for 10 s. The homogenized tissue sample is mixed into the lysis buffer by pipetting 10 times and 100 µl of elution buffer is added to the bottom of each elusion tube.

ASF-specific real-time PCR was performed using a commercial kit (Vet max African swine fever virus detection kit, ThermoFisher, Waltham, MA, USA) validated and certified by the WOAH for ASFV detection (the p72 target gene) [10, 12]. Ct values of <45 denoted positivity.

### 2.4. Detection of Anti-ASFV Antibodies

Whole blood was obtained from ASFV-G-ΔI177L/ΔLVR inoculated animals and their serum was tested for the presence of ASFV antibodies using a commercial ID Screen ASFV competitive ELISA (cELISA; IDvet, rue Louis Pasteur, Grabels, France). Serum was separated from the whole-blood samples and cELISA was performed per the manufacturer's instructions. S/N % was calculated using the following formula:

  $$\begin{array}{c} \text{S/N\%}=\frac{{\text{OD}}_{\text{samples}}-{\text{OD}}_{\text{positive control}}}{{\text{OD}}_{\text{negative control}}-{\text{OD}}_{\text{positive control}}}\times 100. \end{array}$$

Serum samples with S/N% values less than or equal to 40% were considered positive. Serum samples with S/N% greater than or equal to 50% were considered doubtful and repeated.

## 3. Results

### 3.1. Safety of ASFV-G-ΔI177L/ΔLVR in Pregnant Sows

The farrowing performances of the vaccinated and unvaccinated groups of pregnant sows were compared after ASFV-G-ΔI177L/ΔLVR vaccination. Both vaccinated and unvaccinated groups not only maintained normal temperature (Figure 1) and remained clinically healthy until the time of delivery. The piglets were categorized into stillbirth, culling, and onset of lactation. The total number of piglets did not differ within the margin of error in the control and vaccine groups. Three stillbirths were identified out of 22 litters of the sows in the control group. Only two stillbirths and one culling were identified out of the 50 litters of the three vaccinated sows (Table 4). Table S4 compares the results of the recent sow experiment with the previous parity records (total number of litters, number of stillbirths, culling, and number of piglets at the start of lactation) of each sow. It shows that the recent parity performance is within the standard deviation of the previous parity.

### 3.2. Evaluation of ASF Vaccine DNA (ASFV P72 Gene) and Antibodies in Pregnant Sows After ASFV-G-ΔI177L/ΔLVR Vaccination

The sows with late pregnancies were vaccinated with ASFV-G-ΔI177L/ΔLVR at a titer of 10^3.5^ TCID_50_/mL intramuscularly (1 mL). Colostrum, whole blood, and oral and rectal swabs were collected on Day 11 (11 dpv) before farrowing and on Day 18 (18 dpv) at the end of farrowing to detect the ASFV P72 gene, as well as antibodies. First, the P72 ASF target gene was detected in the colostrum samples of three vaccinated sows. Whole blood tested positive for V-1 and V-3 on Days 11 and 18 after vaccination. The V-2 sow was only positive on Day 18. The oral swab samples were positive on Day 18 for V-1 and V-2. In the rectal sample, only the V-1 sow was positive on both Days 11 and 18 of vaccination. The colostrum of V-1 and V-3 had high levels of the ASFV P72 gene immediately after farrowing, but the level was lower in V-2 (Table 5). The antibody concentrations were evaluated in all the samples using a commercial cELISA to detect antibodies in the pregnant sows. The antibody levels in the colostrum of all three sows were high on Day 18 of vaccination (18 dpv). ASF antibodies were detected in the sera of two sows on Day 11 and in all three sows on Day 18 (Table 6).

### 3.3. Detection of the ASFV P72 Gene and Antibodies in the Blood of Piglets After Colostrum Intake

First, piglets that did not consume colostrum, including the control group, did not have the ASFV P72 gene across all the groups. However, the results were not consistent in the piglets that consumed colostrum; three out of five piglets in the V-1 group tested positive for the ASFV P72 gene (60%), two out of five piglets in the V-3 group did (40%), while all of them were negative in the V-2 group (Table 7). The results of the test to detect vaccine antibodies in the blood of the same piglets before and after colostrum intake showed that none of the piglets without colostrum intake, including the control group, had ASF antibodies. However, antibodies were detected in four out of five piglets (80%) in each of the V-1 and V-2 groups and in three out of five piglets (60%) in the V-3 group (Table 8). The detection rate of the antibody was higher than that of the ASFV P72 gene in piglets that consumed colostrum.

### 3.4. Pathologic Findings and Detection of ASF Vaccine DNA (ASFV P72 Gene) in Piglets

Necropsy reports of the four piglets that did not ingest colostrum revealed no significant pathological lesions. Additionally, 10 organs were collected from each piglet, including the submandibular, inguinal, gastrohepatic, and mesenteric lymph nodes; tonsils; liver; heart; lungs; kidneys; and spleen. Detection of the ASFV P72 gene in these tissues was performed using a qPCR assay. The results for the submandibular and gastrohepatic lymph nodes and tonsils were consistent, with the ASFV P72 gene detected in three out of four animals. The genome detection rates were relatively consistent for the remaining organs in two out of four animals. The organ-specific ASFV P72 gene levels were the highest (Ct < 19.9) in the spleen (Table 3).

## 4. Discussion

Since ASF was first reported in 1921, several researchers have attempted to develop various forms of vaccines to eradicate it. These include inactivated vaccines, subunit vaccines, vector vaccines, and LAVs, but most of them have made little progress [14–18]. However, LAVs arguably have the greatest potential for field application. The most effective vaccine candidates against currently circulating strains of ASFV are various forms of LAVs developed by deleting specific virulence-associated viral genes [8, 19, 20]. ASFV-G-ΔI177L, the most well-known of these gene-deleted LAVs with proven safety and efficacy, is the most likely to be commercialized. It was approved for commercialization in Vietnam in July 2023, the first in the world [11]. However, vaccines using the ASFV-G-ΔI177L strain have the disadvantage of only being able to efficiently replicate in primary porcine macrophage cultures. The recent issue of revertant pathogenicity has also resulted in a Notice of withdrawal of select agent regulatory exclusion for ASFV-G-ΔI177L from the United State Department of Agriculture, Animal and Plant Health Inspection [21].

The WOAH standards for the safety of LAVs are becoming more stringent. The safety of vaccines, especially in sows, is becoming increasingly important [13]. Over the past few years, many researchers have reported on the evaluation of various LAV candidates [14]. Among these vaccine candidates, the ASFV-G-ΔI177L/ΔLVR candidate has been demonstrated to be more effective than other LAVs in PIPEC cells in line culture [9]. In this study, we present more promising results for ASF vaccine development. These are based on the details of clinical symptoms, vaccine antibodies, detection of ASFV vaccine DNA (ASFV P72 gene) in blood, and vertical transmission to offspring using the ASFV-G-ΔI177L/ΔLVR vaccine candidate for the first time globally, albeit with limited pregnant sows.

The safety standard of LAV for the prevention of ASF is based on the protection of sows. The reproductive performance of sows is likely one of the most important indicators to evaluate the efficacy of the ASF vaccine and various types of swine disease vaccines, such as those for classical swine fever (CSF), PRRSV, and PCV-2 [22, 23]. In this study, we detected maternal transfer antibodies and the ASFV P72 gene in piglets after the vaccination of pregnant sows, along with asymptomatic sows, robust antibody response, and normal farrowing without abortion.

The performance of late-pregnancy sows treated with the ASFV-G-ΔI177L/ΔLVR vaccine was compared with that of the nonvaccinated control group: Three stillbirths (13.6%) were identified from 22 total litters in the control group, but only two stillbirths (4%) were identified from 50 total litters in the vaccinated group. The total number of stillbirths was more than three times as low despite having more than double as many offspring. This suggests that the vaccine candidate virus had no significant effect on sow performance during late pregnancy and the stillbirths were unrelated to vaccination, thus, providing a preliminary confirmation of the safety of the ASFV-G-ΔI177L/ΔLVR vaccine (Table S4 and Table 4). A recent study on the ASFV vaccine strain ASFV-G-ΔI177L demonstrated reversion to virulence, as evidenced by one of two inoculated pregnant sows developing encephalopathy, a stillbirth rate of 43% among piglets, and a survival rate of only 17%. These findings highlight considerable reproductive safety concerns associated with this strain [24]. In contrast, the currently applied ASFV-G-ΔI177L/ΔLVR vaccine strain appears to present a more favorable safety profile, suggesting improved attenuation and reduced risk of adverse reproductive outcomes.

During the vaccination trial, whole blood, oral, rectal, and colostrum samples were collected and analyzed using qPCR for the presence of the ASFV P72 gene. The pregnant sow V-1 had the P72 gene detected at 11 DPV and 18 DPV in the oral cavity, rectum, and whole blood, with particularly high levels (Ct < 17.0) in whole blood. In the remaining two sows (V-2 and V-3) the P72 gene could only be detected in whole blood, while the results of the oral and rectal samples were not consistent. In addition, relatively high levels of the P72 gene (Ct < 22.5–26.9) were detected in the vaccinated sows V-1 and V-3, while relatively low levels of the P72 gene were detected in V-2. As expected, no ASFV P72 gene was detected in any of the prevaccination body fluid samples (Table 5). The cELISA antibody detection in the vaccinated sows was confirmed in the serum and colostrum. This was consistent with the findings in the V-1 and V-3 sows, with high levels of the P72 gene detected at 11 DPV and 18 DPV (Table 6).

Previous studies have reported abortion induced by a low-virulence strain of ASFV, which was detectable in the fetal placenta, amniotic fluid, and fetal blood [25]. In this study, there was no effect on births in the vaccinated sows. However, the detection of high levels of the ASFV P72 gene and vaccine antibodies, especially in the blood, is expected to provide sufficient data to support the safety of the vaccine in clinical trials to prevent common swine diseases. Furthermore, pharmacokinetics testing of the vaccine to assess immunogenicity is expected to provide important proof-of-concept information to support clinical development. In addition, the detection of high levels of the P72 gene and antibodies in colostrum is consistent with postvaccine maternal transfer of antibodies, a condition for passive immunity, in other common swine diseases. However, the protection against highly virulent field ASFV has not yet been demonstrated.

To further confirm vertical transmission, ASFV P72 gene detection and cELISA antibody detection in the whole blood and serum of piglets before and after colostrum ingestion showed that 60% of piglets in Group V-1 that ingested colostrum had the P72 gene (Ct < 27.5–29.3). Forty percent of the piglets in Group V-3 had it, but none in Group V-2 had it (Ct < 38.4). Notably, the V-2 sow had a very low level of ASFV P72 gene (Ct < 38.4) in the colostrum collected immediately after parturition. All piglets that consumed colostrum from this sow tested negative for the ASFV P72 gene (Table 7). There were relatively high levels of antibodies (80%) in the piglets from sows V-1 and V-2. The level of antibodies was 60% in the piglets from V-3 (Table 8). The ASFV P72 gene was not detected at all in the whole blood of piglets from the V-2 sow, but relatively high levels of antibody were detected in the serum. These two contradictory phenomena are due to vaccine–virus–host interaction and complex immunological phenomena, such as immune resistance inhibiting viral replication or antibody production inhibiting infection by evading immune surveillance, cannot be excluded. On the other hand, transplacental transmission of ASFV has been demonstrated in several papers. High levels of ASFV were present in the tonsils, lymph nodes, and spleen of infected sows, but the transmission of the virus to the fetus is very limited [26, 27].

In addition, ASFV has been reported to be transmitted via artificial insemination, with a replicating virus identified in gilt embryos infected with the virus, as well as fertilized sow fetuses [28, 29]. Therefore, the P72 gene was detected in the major organs of the piglets, even if the whole blood of those without colostrum after ASFV-G-ΔI177L/ΔLVR (ASF-LAV) vaccination was negative or false-positive for the ASFV P72 gene. This confirmed that placental transmission of low-pathogenic ASFV or high-pathogenic ASFV is possible. However, further studies need to determine whether maternally derived antibodies in colostrum are protective against ASFV. For CSF and PRRSV, piglets receiving colostrum from sows vaccinated with LAVs have been shown to have maternally derived antibodies that interfere with their efficacy in piglets [22, 23]. This implies that interference of maternally derived antibodies may not prevent the pathogen from invading the piglet even if sufficient maternally derived antibodies are transferred. Therefore, further study on the defense of ASF in piglets after birth is essential.

In summary, we evaluated the safety of the ASFV-G-ΔI177L/ΔLVR vaccine in pregnant sows at the end of gestation. Regarding safety in pregnant sows, all vaccinated animals remained clinically healthy and exhibited no adverse effects on reproductive performance, as evidenced by normal parturition without any signs of abortion or miscarriage. The findings from this study on piglets indicate that robust ASFV-specific antibodies and the ASFV P72 gene were detected in piglets following colostrum intake, confirming successful maternal antibody transfer. However, while these results confirm the immunogenic response to vaccination in pregnant sows, further controlled challenges studies would be required conclusively establish the protective efficacy of maternal antibodies against ASFV infection in piglets. Despite these encouraging findings, several important safety considerations and limitations must be acknowledged. First, the small sample size, short monitoring period, and limited number of pregnant sows evaluated constrain our ability to detect rare or delayed adverse events. Second, since the study was conducted under controlled experimental conditions within a high-biosecurity laboratory (ABSL-3), it is uncertain whether similar safety outcomes would be observed under field conditions, where stress, coinfections, and diverse management practices may alter the vaccine's behavior. Third, concerning the risk of reversion to virulence, previous studies have shown that the parental ASFV-G-ΔI177L strain can cause severe disease and high piglet mortality in pregnant sows [24]. Although the ASFV-G-ΔI177L/ΔLVR vaccine strain did not exhibit these effects in our study, its genetic stability must be confirmed through long-term passaging and sequencing. Fourth, regarding vertical transmission dynamics, the detection of the ASFV P72 gene in some piglets possibly due to colostrum exposure raises the possibility of intrauterine transmission, which cannot be completely excluded without further histopathologic and molecular investigations.

## 5. Conclusions

The ASFV-G-ΔI177L/ΔLVR live-attenuated vaccine proved safe and immunogenic in late-term pregnant sows. Vaccinated sows carried to term and delivered normally, with no miscarriages, abortions, or other adverse effects observed. Although this small trial did not include a challenge with a virulent field strain, the sows developed a robust ASFV antibody response, suggesting potential protection against wild-type virus. Importantly, maternal immunity was evident in the offspring: most piglets nursing from vaccinated sows had robust ASFV antibodies in their blood and ASFV DNA (the P72 gene) was detected in their samples. This indicates that protective antibodies were transferred through colostrum (passive transfer) and confirms transplacental (vertical) transmission of the vaccine virus to fetuses without including detecting detectable adverse effects. Despite these promising results, the study's scope was limited—it was a preliminary, small-scale trial and the first of its kind in pregnant sows. Further large-scale, long-term field studies are required to fully evaluate the vaccine's safety and efficacy, including viral shedding and stability, vertical transmission risk, and maternal antibody interference.

## Figures and Tables

**Table 1:** ASF live attenuated vaccine vaccination status of pregnant sows by group.

Group	Number of pregnant sow	Gestation period (day)	Vaccine	Titer	Route
Group V (vaccinated)	3	90–95	ASFV-G-∆I177L/∆LVR	10^3.5^ TCID_50_	IM
Group C (nonvaccinated)	2	90–95	DMEM	—	IM

Abbreviation: DMEM, Dulbecco's modified eagle's medium.

**Table 2:** Labor induction injection and birth status.

Group	Labor induction (dose/mL)	Date of labor induction inoculation	Date of delivery completion	Birth rate (individual)
V-1 (888)	2 mL	18 DPV	20 DPV	16
V-2 (779)	2 mL	18 DPV	20 DPV	15
V-3 (797)	2 mL	18 DPV	20 DPV	19
C-1 (574)	2 mL	18 D	20 D	13
C-2 (988)	2 mL	18 D	20 D	9

*Note:* V: ASFV-G-ΔI177L/ΔLVR vaccinated; (): parentheses is sow's individual number; C: nonvaccinated control; DPV (V groups): day postvaccination; D (C groups): day.

**Table 3:** Detection of ASFV P72 gene in organ samples from piglets before intake of colostrum.

Organs	ASFV genome copy (qPCR, Ct value^a^)			
	V1 sow's offspring			
	V1-1	V1-2	V1-3	V1-4
Submandibular lymph node	35.7	30.9	30.5	45.0
	35.0	31.1	30.2	45.0

Inguinal lymph node	45.0	31.8	33.1	45.0
	45.0	29.7	33.4	45.0

Gastrohepatic lymph node	37.4	27.0	35.9	45.0
	45.0	27.0	35.2	45.0

Mesenteric lymph node	45.0	29.7	34.7	45.0
	45.0	30.0	34.1	45.0

Tonsil	45.0	31.9	35.4	45.0
	38.3	31.3	33.9	45.0

Liver	45.0	20.6	33.2	45.0
	45.0	20.7	33.3	45.0

Heart	45.0	31.5	37.3	45.0
	45.0	30.8	45.0	45.0

Lung	38.6	25.5	45.0	45.0
	36.8	25.7	45.0	45.0

Kidney	45.0	29.9	45.0	45.0
	45.0	29.7	45.0	45.0

Spleen	45.0	19.9	36.4	45.0
	45.0	20.1	38.1	45.0

^a^Ct value <45 was considered positive.

**Table 4:** Farrowing record of ASFV-G-ΔI177L/ΔLVR vaccinated and nonvaccinated sows.

Group	Previous birth parity record/number of birth	^a^Present birth parity record/number of birth	^a^Still birth	^a^Culling (weak born)	^a^Crushing	^a^Onset of lactation
V-1 (888)	5^th^/20	6^th^/16	1	—	—	15
V-2 (779)	5^th^/13	6^th^/15	—	—	—	15
V-3 (797)	4^th^/20	5^th^/19	1	1	—	17
C-1 (574)	6^th^/14	7^th^/13	1	—	—	12
C-2 (988)	5^th^/13	6^th^/9	2	—	—	7

*Note:* (): Parentheses are sow's individual number. V: ASFV-G-ΔI177L/ΔLVR vaccinated; C: nonvaccinated control.

^a^Indicates recent parity record, the 5^th^, 6^th^, and 7^th^ indicates recent parity.

**Table 5:** Evaluation of ASFV P72 gene by body fluid after ASFV-G-ΔI177L/ΔLVR vaccination.

Group	Real-time PCR (Ct^a^)				
	Sample collection (D/DPV)	WB (whole blood)	Oral	Rectal	Colostrum
V-1 (888)	11 DPV	17.0	41.1	30.9	—
	18 DPV	17.4	37.8	30.6	22.5

V-2 (779)	11 DPV	45.0	45.0	45.0	—
	18 DPV	35.5	39.7	45.0	38.4

V-3 (797)	11 DPV	21.2	45.0	45.0	—
	18 DPV	23.7	45.0	45.0	26.9

C-1 (574)	11 D	45.0	45.0	45.0	—
	18 D	45.0	45.0	45.0	45.0

C-2 (988)	11 D	45.0	45.0	45.0	—
	18 D	45.0	45.0	45.0	45.0

^a^Ct value <45 was considered positive.

**Table 6:** ASF vaccine antibody test in serum and colostrum in pregnant sows.

Group	cELISA (S/N^a^)		
	Sample collection (D/DPV)	Serum	Colostrum
V-1 (888)	11 DPV	18.6	—
	18 DPV	11.1	12.6

V-2 (779)	11 DPV	54.8	—
	18 DPV	10.3	27.3

V-3 (797)	11 DPV	20.6	—
	18 DPV	27.5	22.3

C-1 (574)	11 D	76.3	—
	18 D	71.8	89.7

C-2 (988)	11 D	77.1	—
	18 D	76.3	87.2

^a^S/N (%): Less than or equal to 40% are considered positive.

**Table 7:** Detection of ASFV 72 gene in piglet before and after uptake of colostrum.

Division	Animal ID	ASFV genome copy (qPCR, Ct value^a^)				
		Test (vaccinated pregnant) group			Control group	
		V-1 (888)	V-2 (779)	V-3 (797)	C-1	C-2
Before colostrum intake	1	45.0	45.0	45.0	45.0	45.0
	2	36.7	45.0	45.0	45.0	45.0
	3	45.0	45.0	45.0	45.0	45.0
	4	45.0	45.0	45.0	45.0	45.0
	5	45.0	45.0	45.0	45.0	45.0

After colostrum intake	1	45.0	45.0	45.0	45.0	–^b^
	2	27.4	45.0	45.0	45.0	—
	3	45.0	45.0	45.0	45.0	—
	4	29.3	45.0	22.4	45.0	—
	5	27.5	45.0	15.9	45.0	—

^a^Ct value <45 was considered positive.

^b^“–”: Not tested.

**Table 8:** Detection of ASF antibody in piglet before and after uptake of colostrum.

Division	Animal ID	ASFV antibody (cELISA, S/N %^a^)				
		Test (vaccinated pregnant) group			Control group	
		V-1 (888)	V-2 (779)	V-3 (797)	C-1	C-2
Before colostrum intake	1	90.8	90.4	85.8	97.2	94.1
	2	93.4	93.5	86.4	94.9	97.2
	3	98.9	91.9	87.6	97.2	99.4
	4	98.1	88.9	80.8	95.4	97.8
	5	89.3	89.7	87.4	96.9	94.9

After colostrum intake	1	8.6	91.4	40.1	100.2	–^b^
	2	8.3	20.9	31.7	91.7	—
	3	12.8	19.6	75.9	85.5	—
	4	8.4	21.7	35.1	79.6	—
	5	66.9	20.9	35.0	86.2	—

^a^S/N %: Less than or equal to 40% are considered positive.

^b^“−”: Not tested.

## Data Availability

The data and questionnaires that support the findings of this study are available from the corresponding author upon reasonable request.

## Supporting Information

Supporting Information Table S1. ELISA test for prescreening of major swine diseases. Table S2. PCR test for prescreening of major swine diseases. Table S3. Antigen (PCR) and antibody (ELISA) tests to identify major diseases in pregnant sows prior to ASF vaccine trials. Table S4. Description of detailed records of farrowing performance from the first pregnancy of each sow to before the ASF vaccine experiment.
