# Supplementary material for: Safety of Live Attenuated ASFV-G-ΔI177L/ΔLVR Vaccination in Sows With Advanced Pregnancies
Source: Transbound Emerg Dis. 2025 Jul 13;2025:8007143. doi: 10.1155/tbed/8007143 (PMC12277044; doi:10.1155/tbed/8007143)
Supplement: Supporting Information — Table S1. ELISA test for prescreening of major swine diseases. Table S2. PCR test for prescreening of major swine diseases. Table S3. Antigen (PCR) and antibody (ELISA) tests to identify major diseases in pregnant sows prior to ASF vaccine trials. Table S4. Description of detailed records of farrowing performance from the first pregnancy of each sow to before the ASF vaccine experiment. [file 8007143.f1.docx]

**Supplementary Tables**

Table S1. ELISA test for pre-screening of major swine diseases.

| **Infectious**  **Agent** | **CAT.**  **Number** | **Product Name**  **(Website linked)** | **Manufacturer**  **(Website linked)** |
| --- | --- | --- | --- |
| ASFV | ASFC | [ID Screen^Ⓡ^ African Swine Fever Competition](https://www.innovative-diagnostics.com/produit/id-screen-african-swine-fever-competition/) | [Innovative Diagnostics](https://www.innovative-diagnostics.com/about-us/) |
| PRRSV | 99-18070 | [IDEXX PRRS X3 Ab Test](https://www.idexx.co.uk/en-gb/livestock/livestock-tests/swine-tests/idexx-prrs-x3-ab-test-worldwide/) | [IDEXX](https://www.idexx.co.uk/en-gb) |
| FMDV | 7610440 | [PrioCHECK™ FMDV NS Antibody ELISA Kit](https://www.thermofisher.com/order/catalog/product/7610440) | ThermoFisher Scientific |
| `PCV2 | SK105 | [PCV2 ELISA](https://www.biochek.com/swine-elisa/porcine-circovirus-type-2-antibody-test-kit/) | [BioChek](http://www.biocheck.com/) |
| CSFV | SK106 | CSFV E2 [ELISA](https://www.biochek.com/swine-elisa/classical-swine-fever-antibody-test-kit/) | [Biochek](http://www.biocheck.com/) |
| Mycoplasma | 99-06733 | [IDEXX M. hyo Ab Test](https://www.idexx.com/en/livestock/livestock-tests/swine-tests/idexx-m-hyo-ab-test/) | [IDEXX](https://www.idexx.co.uk/en-gb) |

Table S2. PCR test for pre-screening of major swine diseases.

| **Infectious**  **Agent** | **CAT.**  **Number** | **Product Name**  **(Website linked)** | **Manufacturer**  **(Website linked)** |
| --- | --- | --- | --- |
| ASFV | A28809 | [VetMAX African Swine Fever Virus Detection Kit](https://www.thermofisher.com/order/catalog/product/A28809) | ThermoFisher Scientific |
| PRRSV | A35751 | [VetMAX™ PRRSV EU & NA 2.0 Kit](https://www.thermofisher.com/order/catalog/product/A35751) | ThermoFisher Scientific |
| FMDV | NM-FMD-31 | [VDx® FMDV 3Diff/PAN qRT-PCR set](https://www.mediandiagnostics.com/en/page/21_view?idx=87) | [MEDIAN Diagnostics](https://www.mediandiagnostics.com/en/) |
| PCV2 | A28809 | [VetMAX™ Porcine PCV2 Quant Kit](https://www.thermofisher.com/order/catalog/product/QPCV) | ThermoFisher Scientific |
| CSFV | NS-CSF-31 | [VDx® CSFV qRT-PCR > PCR](https://www.mediandiagnostics.com/en/page/21_view?idx=48) | [MEDIAN Diagnostics](https://www.mediandiagnostics.com/en/) |
| Mycoplasma | 4460626 | [MycoSEQ™ Mycoplasma Detection Kits](https://www.thermofisher.com/order/catalog/product/kr/ko/4460626) | ThermoFisher Scientific |

Table S3. Pre-screening test for differential diagnosis of major swine diseases in sows.

| Infectious Agent | Ag*(qPCR) | | | | | Ab**(ELISA) | | | | |
| --- | --- | --- | --- | --- | --- | --- | --- | --- | --- | --- |
|  | V-1 | V-2 | V-3 | C-1 | C-2 | V-1 | V-2 | V-3 | C-1 | C-2 |
| ASFV | - | - | - | - | - | - | - | - | - | - |
| PRRSV | - | - | - | - | - | - | + | + | + | - |
| FMDV | - | - | - | - | - | + | + | + | + | + |
| PCV2 | - | - | - | - | - | + | + | + | + | + |
| CSFV | - | - | - | - | - | + | + | + | + | + |
| Mycoplasma | - | - | - | - | - | + | - | - | + | - |

*Ag: antigen, **Ab: antibody, -: Negative, +: Positive

Table S4. Pre-farrowing average record of sows used in ASF vaccine candidate trials.

| Group | Sow parity | Total Farrowing | Stillbirth | Culling | Crushing | Onset of Lactation |
| --- | --- | --- | --- | --- | --- | --- |
| V-1 (888) | 1^st^~5^th^ | 13.60±5.13 | 0.40±0.89 | 3.80±3.03 | 0.00±0.00 | 0.00±0.00 |
| V-2 (779) | 1^st^~5^th^ | 15.00±4.06 | 0.80±1.30 | 2.80±2.49 | 0.00±0.00 | 11.40±1.34 |
| V-3 (797) | 1^st^~4^th^ | 13.50±5.45 | 0.25±0.50 | 3.25±3.59 | 0.00±0.00 | 10.00±2.45 |
| C-1 (574) | 1^st^~6^th^ | 12.17±2.64 | 0.67±1.21 | 1.67±1.75 | 0.67±1.03 | 9.17±1.47 |
| C-2 (988) | 1^st^~5^th^ | 14.40±4.62 | 1.20±0.84 | 2.60±1.82 | 0.00±0.00 | 10.20±2.77 |

(): Parentheses are sow’s individual number, The 4^th^, 5^th^ & 6^th^ indicates past parity record
